# *Rickettsia australis* Activates Inflammasome in Human and Murine Macrophages

**DOI:** 10.1371/journal.pone.0157231

**Published:** 2016-06-30

**Authors:** Claire Smalley, Jeremy Bechelli, Dedeke Rockx-Brouwer, Tais Saito, Sasha R. Azar, Nahed Ismail, David H. Walker, Rong Fang

**Affiliations:** 1 Department of Pathology, University of Texas Medical Branch at Galveston, Galveston, Texas, United States of America; 2 Department of Pathology, University of Pittsburgh, Pittsburgh, Pennsylvania, United States of America; Upstate Medical University, UNITED STATES

## Abstract

Rickettsiae actively escape from vacuoles and replicate free in the cytoplasm of host cells, where inflammasomes survey the invading pathogens. In the present study, we investigated the interactions of *Rickettsia australis* with the inflammasome in both mouse and human macrophages. *R*. *australis* induced a significant level of IL-1β secretion by human macrophages, which was significantly reduced upon treatment with an inhibitor of caspase-1 compared to untreated controls, suggesting caspase-1-dependent inflammasome activation. *Rickettsia* induced significant secretion of IL-1β and IL-18 *in vitro* by infected mouse bone marrow-derived macrophages (BMMs) as early as 8–12 h post infection (p.i.) in a dose-dependent manner. Secretion of these cytokines was accompanied by cleavage of caspase-1 and was completely abrogated in BMMs deficient in caspase-1/caspase-11 or apoptosis-associated speck-like protein containing a caspase activation and recruitment domain (ASC), suggesting that *R*. *australis* activate the ASC-dependent inflammasome. Interestingly, in response to the same quantity of rickettsiae, NLRP3^-/-^ BMMs significantly reduced the secretion level of IL-1β compared to wild type (WT) controls, suggesting that NLRP3 inflammasome contributes to cytosolic recognition of *R*. *australis in vitro*. Rickettsial load in spleen, but not liver and lung, of *R*. *australis*-infected NLRP3^-/-^ mice was significantly greater compared to WT mice. These data suggest that NLRP3 inflammasome plays a role in host control of bacteria *in vivo* in a tissue-specific manner. Taken together, our data, for the first time, illustrate the activation of ASC-dependent inflammasome by *R*. *australis* in macrophages in which NLRP3 is involved.

## Introduction

Rickettsial infections pose serious public health problems because of their potential to cause life-threatening human infection and to be used as biological weapons, a situation that is exacerbated by the lack of a Food and Drug Administration-approved vaccine [1]. Rickettsiae are obligately intracellular bacteria which possess the ability to quickly escape phagosomal vacuoles and replicate within the cytosol of host cells. However, the interactions of rickettsiae with cytosolic sensors, such as nucleotide binding and oligomerization domain (NOD)-like receptors (NLRs) in innate immune cells, have never been investigated. This is a gap in our knowledge that impedes the development of new therapeutic approaches and vaccine development strategies.

The inflammasome is a large multi-protein complex consisting of NLRs and the protease, caspase-1 [2]. Inflammasome activation by pathogens hinges upon violation of the host cell cytosol by activities such as those of pore-forming toxins, specialized microbial secretion systems, or the cytosolic presence of the pathogen itself [2]. In response to these stimulants and/or danger signals (e.g., ATP), activation of NLRs can oligomerize ASC, which in turn activates caspase-1 to trigger its protease activity. Caspase-1 then mediates cleavage of pro-IL-1β and pro-IL-18 and secretion of IL-1β and IL-18 and/or inflammatory cell death, known as pyroptosis [3]. Among NLRs that have been described as critical components of inflammasomes, NLRP3 plays a critical role in adjuvant-driven cellular immunity and, as such, exploitation of this pathway by vaccines may enhance efficacy, thus reinforcing the importance of investigating inflammasome activation and understanding the underlying mechanisms [4].

By using murine models of rickettsioses, we have identified the critical roles of IFN-γ, dendritic cells (DCs), NK cells, TLRs and effector CD8^+^ T cells in host protective immunity against rickettsial infection [5–12]. Although *R*. *conorii*-infected C3H/HeN mice provide an excellent mouse model of human rickettsial infection [13], we utilized *R*. *australis*-infected C57BL/6 (B6) mice in the present study because of the availability of various gene-deficient mice on the B6 background. *Rickettsia australis* is the etiologic agent of Queensland tick typhus [14]. Infection of B6 mice with *R*. *australis* provides an excellent murine model of rickettsial disease that targets endothelial cells (ECs) and macrophages and mimics the pathological findings of spotted fever group (SFG) rickettsioses in humans [15–18]. Using this model, we investigated whether rickettsiae are recognized in the cytosol by inflammasome and the mechanisms involved *in vitro* and *in vivo*.

Although ECs are the primary target cells for rickettsial infection, pathogenic rickettsiae also invade macrophages as observed in established animal models and in the arthropod feeding inoculation site [19, 20]. In response to IFN-γ and TNF-α, macrophages are activated and serve as crucial effector cells mediating clearance of intracellular pathogens. Upon infection, perivascular infiltration of macrophages, together with lymphocytes and other cells, is a component of rickettsial vasculitis [21]. Therefore, understanding the interactions of rickettsiae with macrophages will greatly increase our knowledge regarding the pathogenesis of rickettsial infections and immunity against rickettsiae. In the present study, we focused on interactions of inflammasome with *R*. *australis* in mouse and human macrophages. We hypothesize that *R*. *australis* are recognized by cytosolic sensors, ASC-dependent inflammasome involving NLRP3, in macrophages leading to secretion of IL-1β and IL-18.

## Materials and Methods

### Rickettsia

*Rickettsia australis* (Cutlack strain) were cultivated in Vero cells and purified as previously described with modifications [6, 22]. Briefly, infected cells were collected and suspended in SPG buffer (218 mM sucrose, 3.76 mM KH_2_PO_4_, 7.1 mM K_2_HPO_4_, 4.9 mM potassium glutamate) after sonication. The rickettsiae were placed on the top of 32%, 26% and 20% OptiPrep Density Gradient medium (Sigma- Aldrich, St. Louis, MO) in 6 × SPG bed. After centrifugation, rickettsiae were washed and collected. These stocks were used to infect macrophages *in vitro*. *Rickettsia australis* (Cutlack strain) used for animal inoculation were cultivated in specific pathogen free embryonated chicken eggs. Yolk sacs from infected eggs were homogenized in a Waring blender and diluted to a 10% suspension in SPG buffer. All of these rickettsial stocks were quantified by plaque assay before use in experiments, as previously described [15]. The rickettsial stock was stored at -80°C until use. All the experiments described in this study were performed in a certified biosafety level 3 (BSL3) laboratory at UTMB.

### Generation of human macrophages

THP-1 cells were purchased from ATCC and cultured as previously described without antibiotics [23]. THP-1 cells were differentiated in 100 μg/μl PMA (Sigma-Aldrich), reconstituted in DMSO (Sigma-Aldrich) for 16 h, followed by 24 h recovery in fresh medium. Cells were plated at a density of 1 × 10^6^ cells per well in a 6-well-plate, and infected with *R*. *australis* at an MOI of 5.

Human peripheral blood monocytes (PBMC) were isolated from buffy coats obtained from the UTMB blood bank. Cells were isolated as previously described [24]. Cells were plated at a density of 1 × 10^6^ cells in each well in a 12-well-plate, and infected with *R*. *australis* at an MOI of 2 or 5.

### Confocal immunofluorescence microscopy

For immunofluorescence detection of *R*. *australis* in human PBMC-derived macrophages, cells were first seeded on glass coverslips in 12-well plates one day before infection. At 24 h p.i., cells were washed with PBS, fixed with 4% paraformaldehyde in PBS for 20 min, permeabilized with 0.5% Triton-X in PBS for 20 min and blocked with 3% BSA in PBS for 30 min. Samples were incubated with rabbit polyclonal antibodies directed against *R*. *australis*, goat anti-human CD68 (BioLegend, San Diego CA, #Y1/82A) followed by appropriate secondary antibodies including Alexa Fluor488- conjugated chicken anti-goat IgG (Life Technologies, NY, #A21467). The anti-CD68 antibody (Ab) preferentially labels human macrophages. Nuclei were stained with DAPI in ProLong® Gold Antifade Mountant (Life Technology, NY, P-36931). Coverslips were sealed with nail polish, and visualized by confocal microscopy with a 60 × water immersion lens (Olympus Fluoview 1000) using FV10-ASW software (Olympus, PA).

### Measurements of cytokines

Supernatants of cell cultures were collected and filtered to be rickettsiae-free before removal from the BSL3 laboratory. Cytokine concentrations in the culture supernatant were measured by using Quantikine enzyme-linked immunosorbent assay (ELISA) kits. Detection of cytokines in murine samples were performed using the ELISA kit from eBioscience (San Diego CA). The limits of detection of the cytokines were as follows: IL-1β, 16 pg/ml; IL-18, 25.6 pg/ml; and IL-10, 62.5 pg/ml. Measurement of IL-1β and IL-18 in human samples was performed using ELISA kits from R&D Systems (IL-18, limit of detection: 12.5 pg/ml) and eBioscience (IL-1β, limit of detection: 1pg/ml).

### Caspase-1 inhibition

To determine whether secretion of IL-1β by infected human macrophages is dependent on caspase-1, we employed the irreversible inhibitor specific for caspase-1 (Z-YVAD-FMK, 30 nM) (Enzo Life Sciences, Farmingdale NY). The inhibitor was added to a complete monolayer of THP-1 derived macrophages for 4 hours before infection with *R*. *australis* at an MOI of 5.

### Mice and generation of bone marrow-derived macrophages

Wild type (WT) B6 mice, caspase-1/11-double knockout mice and C57BL/6N (B6N) mice were purchased from Jackson Laboratories (Bar Harbor, Maine). ASC^-/-^ and NLRP3^-/-^ mice were gifts of Dr. Vishva Dixit at Genentech (California, USA). All mice were maintained and manipulated in an animal biosafety level-3 (ABSL3) facility at the University of Texas Medical Branch, Galveston, TX. This study was carried out in strict accordance with the recommendations in the guidelines of the National Institutes of Health Guide for the Care and Use of Laboratory Animals. All experiments and procedures were approved by the Institutional Animal Care and Use Committee (IACUC) and Institutional Biosafety Committee of the University of Texas Medical Branch at Galveston. For *in vivo* experiments, mice were inoculated intravenously (i.v.) through the tail vein with *R*. *australis* at the dose indicated. After infection, mice were monitored daily for signs of illness until day 20 post infection (p.i.). No animal death was observed. Some of the mice were euthanized on days 2 and 4 p.i.. Mice were first anesthetized by inhalational isoflurane (Isoflurane® USP, Piramal Healthcare Limited, 502321 Andhra Pradesh, India) and then euthanized by CO_2_ narcosis and asphyxia followed by cervical dislocation. After the death of animals, mouse tissues including lung, liver and spleen were isolated for evaluation of the bacterial replication and pathology by quantitative real-time PCR and histopathological analysis. All necessary precautions were taken to minimize the discomfort and pain to animals used in the experiments.

Generation of primary bone marrow-derived macrophages (BMMs) from 6–8 week old female WT mice, ASC^-/-^ mice and NLRP3^-/-^ mice was performed as previously described [25]. Briefly, after femurs and tibias were dissected, bone marrows were flushed, and cells were cultivated in low-endotoxin DMEM/F12 containing 10% (v/v) newborn calf serum (Thermoscientific, Gibco, CA, 16010159) supplemented with either 20% supernatant from L929 cell culture or recombinant M-CSF (PeproTech, NJ, 315–02) at 37°C in 5% CO_2_. On day 6 of culture, cells were harvested and characterized by flow cytometric analysis after staining with anti-F4/80 and anti-CD11b antibodies. Cells were used if they contained ≥85 to 90% F4/80 (+) and CD 11b (+) cells. These cells were plated in 24-well plates at a density of 1 × 10^6^ cells/ well in DMEM/F12 containing 10% newborn calf serum and used for experiments within 24 hrs.

### *In vitro* infections

For *in vitro* rickettsial infection, macrophages were infected with *R*. *australis* at a multiplicity of infection (MOI) of 10:1, 6:1, 2:1 or as indicated. To synchronize bacterial internalization, rickettsiae were centrifuged onto the cells at 560 × *g* for 5 min. Cells were incubated continuously at 37°C in 5% CO_2_. Uninfected cells served as negative controls. Positive controls were the cells primed with LPS (200 ng/ml) for 4 hours followed by incubation with ATP (5 mM) for 45 min. The viability of infected mouse BMMs was examined by trypan blue staining and LIVE/DEAD® Fixable Near-IR Dead Cell Stain Kit (Life Technologies, Grand Island, NY) in accordance with the manufacturer’s protocol. Positive control was composed of half of them alive and half of them dead. Flow cytometry was performed on 30,000 cells with a FACSCalibur laser cytometer (Becton-Dickinson, BD Biosciences, San Jose, CA). Data analysis on the entire ungated cell population was performed using FlowJo software.

### Western immunoblotting

For evaluation of the activation of inflammasome, uninfected and infected cells were lysed with RIPA lysis buffer (EMD Millipore, MA, 20–188) supplemented with protease inhibitors (Roche, IN, 05892970001). The soluble part of cell lysates was isolated by centrifugation and used for immunoblotting. Cell culture supernatants were processed by centrifugal filter units (3K) (Amicon) as described by the manufacturer. Briefly, 2 ml of supernatant were loaded onto columns and centrifuged at 7000 × *g* for 60 min at 4°C. Protein concentration was determined by BCA Assay (Pierce Biotechnology). The cell lysates and concentrated supernatant were separated by SDS-PAGE, transferred to a polyvinylidene difluoride (PVDF) membrane and probed with anti-caspase-1 p20 antibody (EMD Millipore, MA, 06-503-I) for lysates, and anti-caspase-1 p10 antibody (sc-514, Santa Cruz Biotechnology) for concentrated supernatants. Immunoreactive bands were visualized using an appropriate secondary antibody and electrochemiluminescence detection reagents (Thermoscientific, Pierce, IL, 32106). Equal protein loading of the gels was controlled by detecting β-actin with mouse monoclonal Ab (Sigma, MO, A1978) in the cellular lysates. The detection of pro-caspase-1 (45 kDa) and activated caspase-1 (10 or 20 kDa) is indicative of activation of inflammasome as described previously [2].

### Quantification of bacterial loads in infected tissues *in vivo* and NLRP3 transcripts in infected macrophages *in vitro* by quantitative real-time PCR

To determine the rickettsial load in infected tissues, mouse lung, liver and spleen were isolated from infected animals. Rickettsial loads were quantified using quantitative PCR following DNA extraction as described in our previous studies with modifications [8, 16]. Briefly, tissues were first placed in RNA later (Thermo Fisher Scientific, Waltham, MA). Total DNA was extracted using a DNeasy tissue kit (Qiagen, CA, 69506), and rickettsial burdens were determined using an iCycler IQ from Bio-Rad (Hercules, CA). The following primers (Sigma-Genosys, St. Louis, MO) and probes (Biosearch Technologies, Novato, CA) targeting *Rickettsia*-specific citrate synthase (CS) gene (*gltA*) as described previously [8, 16] (*gltA* forward, *GAGAGAAAATTATATCCAAATGTTGAT*; *gltA* reverse, *AGGGTCTTCGTGCATTTCTT*; *gltA* probe, *CATTGTGCCATCCAGCCTACGGT*). The results were normalized to the weight of the same sample and expressed as copy number of CS genes per ng of tissue. Tissues from uninfected mice served as a negative control.

Uninfected and *R*. *australis*-infected WT BMMs were collected in RNA later at the indicated time points. Total RNA was prepared using Qiagen RNeasy Mini kit (Valencia, CA) following the manufacturer’s recommendations. Reverse transcription (RT) was performed using isolated and DNase-treated RNA with Bio-Rad iScript cDNA synthesis kit (Hercules, CA). The resulting cDNAs were used as template for quantitative reverse transcription–polymerase chain reaction (RT-PCR). Gene expression of NLRP3 was determined using SYBR Green PCR Master Mix on an iCycler IQ (Bio-Rad, Hercules, CA) using primers targeting NLRP3 (Forward 5’-*CCT TCA GGC TGA TCC AAG AG*-3’, Reverse 5’-*GCC AAA GAG GAA TCG GAC AAC-*3’) and GAPDH (Forward 5’-*ATGGTGAAGGTCGGTGTGAA*-3’, Reverse 5’- *CTCCTTGGAGGCCATGTA*-3’) as described previously [26, 27]. Quantitative results were expressed as the mRNA relative ratio (2-∆∆Ct) normalized to the housekeeping gene as previously described [28].

### Statistical analysis

For comparison of multiple experimental groups, the one-way analysis of variance (ANOVA) with Bonferroni's and Tukey’s procedure was used. Two-group comparison was conducted using either Student *t*-test or Welch's *t*-test depending on whether the variance between two groups was significantly different. When two factors were included in the comparison, two-way ANOVA with Bonferroni post-test was used. For testing the difference in survival between different mouse groups, data were analyzed by the product limit (Kaplan-Meier) method. All the statistical analyses were performed using GraphPad Prism software version 5.01. *P* values of 0.05 or less were the threshold for statistical significance.

## Results

### *R*. *australis* infects human macrophages and activates inflammasome

**Fig 1:**
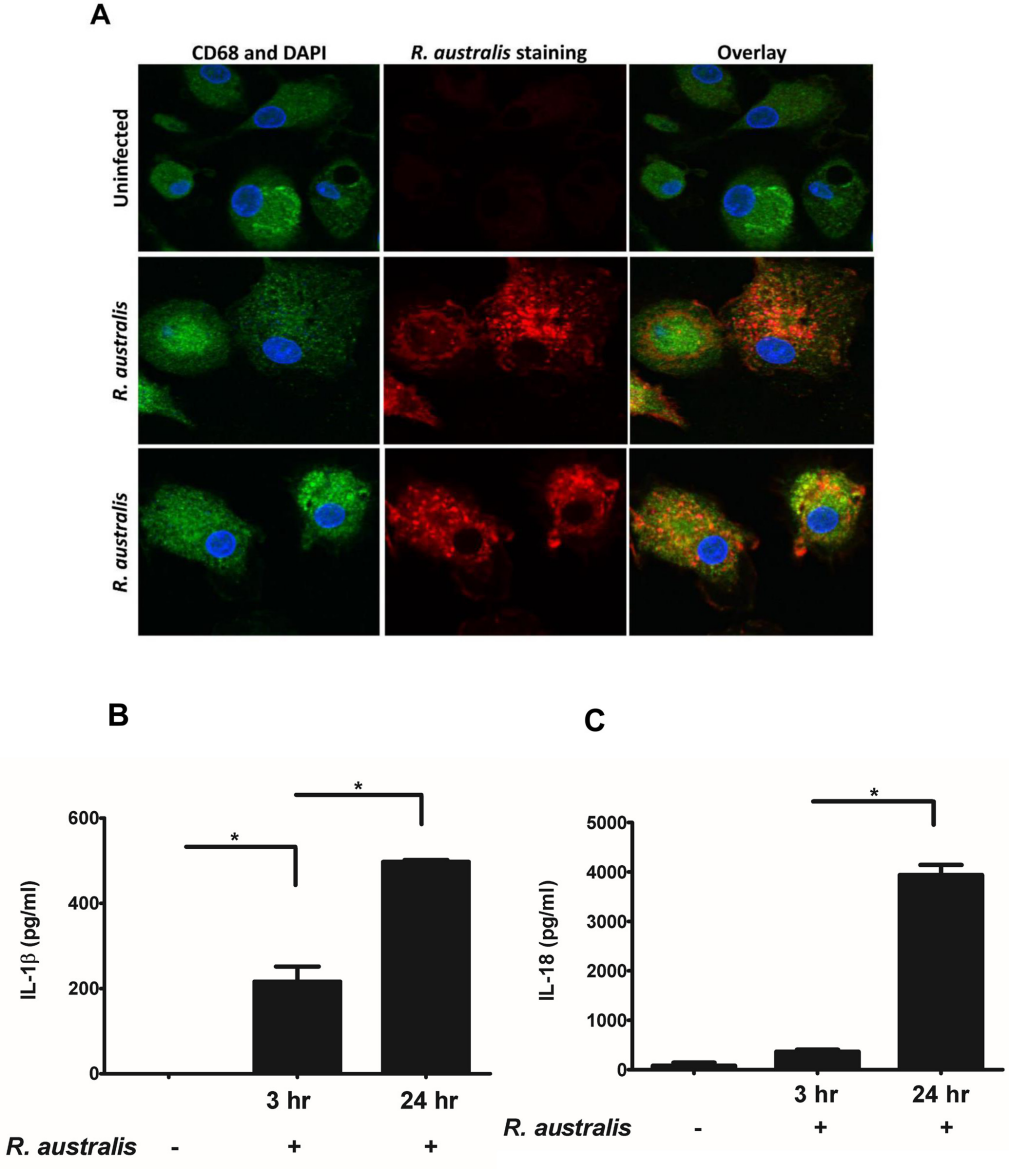
Infection of human PBMC-derived macrophages with *R*. *australis* and activation of inflammasome. Human PBMC-derived macrophages were prepared and infected with *R*. *australis*. Cells and culture supernatant were collected at 24 h p.i.. A, Cytosolic rickettsiae were detected by confocal immunofluorescence microscopy after infection with *R*. *australis* at an MOI of 5. Images were acquired using 60 × magnification with a water immersion lens. Macrophages are depicted in green, nuclei (DAPI) in blue, and *R*. *australis* in red. Secretion levels of IL-1β (B) and IL-18 (C) at 3 h and 24 h p.i. by human PBMC-derived macrophages infected with *R*. *australis* at an MOI of 2 were determined by ELISA. Data represent two independent experiments with consistent results. Each experiment included at least 4 replicates. *, *p*<0.05.

So far, it remains elusive whether human macrophages can be infected by *R*. *australis in vitro*. To investigate the activation of inflammasome by rickettsiae in human macrophages, we first examined whether *R*. *australis* infects PBMC-derived macrophages by confocal immunofluorescence microscopy. *R*. *australis* (red) was detected in the cytosol (nucleus as blue) of infected human PBMC-derived macrophages, suggesting that rickettsiae were effectively taken up by and established infection in these cells (Fig 1A). Interestingly, human PBMC-derived macrophages secreted a significantly higher level of IL-1β as early as 3 h p.i. compared to uninfected controls (Fig 1B). The levels of IL-1β secreted by these infected macrophages were greater at 24 h p.i. compared to 3 h p.i. (Fig 1B). At 24 h p.i., *R*. *australis* also induced a significantly increased level of IL-18 in human PBMC-derived macrophages (Fig 1C). These results suggest that *R*. *australis* infects primary human macrophages and promotes secretion of the inflammasome-derived cytokines including IL-1β and IL-18.

**Fig 2:**
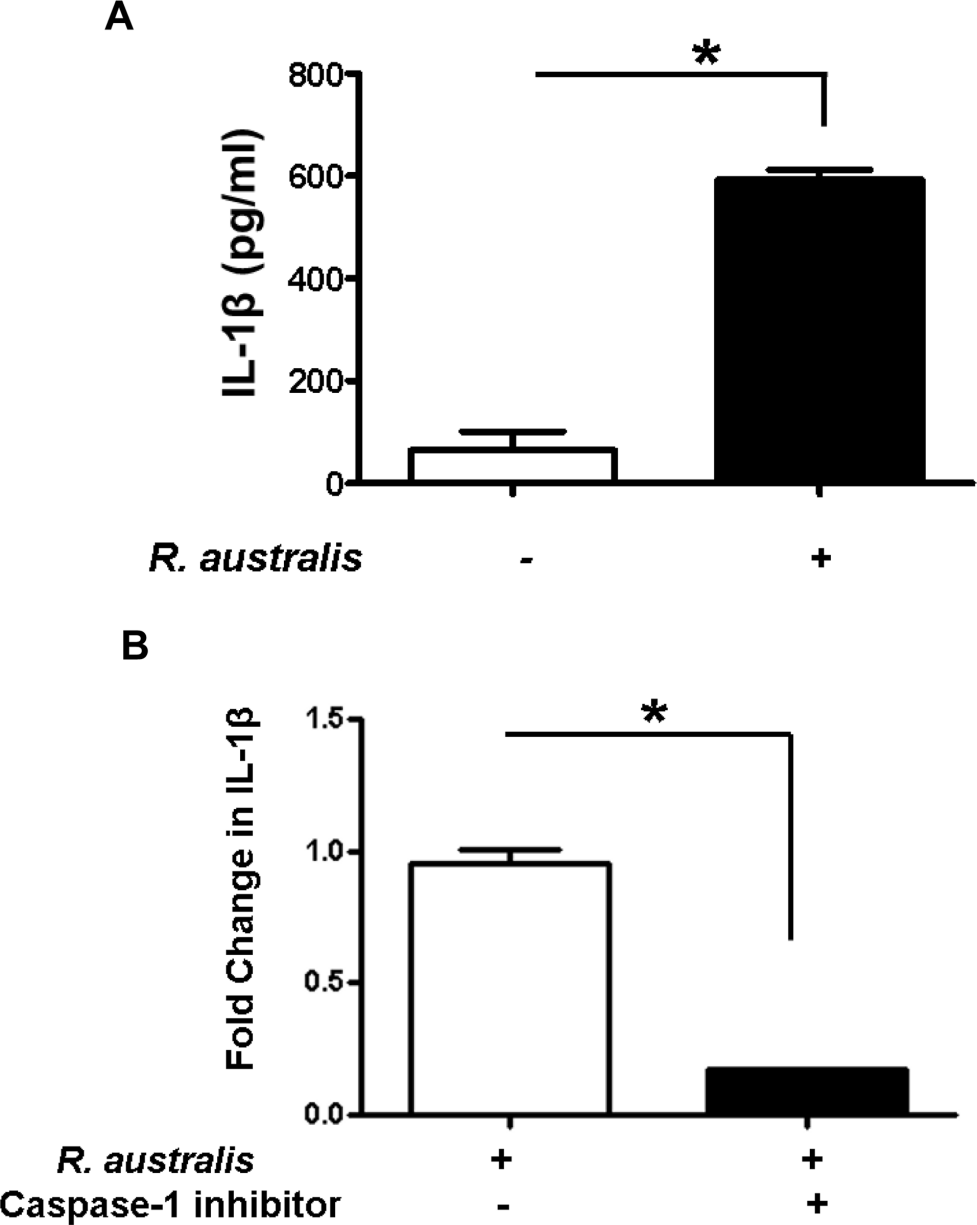
Activation of inflammasome by rickettsiae in THP-1 derived macrophages and caspase-1-dependent secretion of IL-1β. Human THP-1 cells were differentiated to macrophages using PMA, and infected with *R*. *australis* at an MOI of 5. A, Infection with *R*. *australis* induced a significant increase in IL-1β production compared to uninfected cells at 24 h p.i.. B, Inhibition of caspase-1 significantly reduced the secretion levels of IL-1β by *R*. *autralis*-infected THP-1 derived macrophages. The fold change in IL-1β by treated cells vs. untreated controls was calculated and compared. Data represent two independent experiments with consistent results. Each experiment included at least 4 replicates. *, *p*<0.05.

To further study the mechanisms involved in secretion of inflammasome-derived IL-1β in human macrophages, we examined the dependence of IL-1β secretion on caspase-1 in *R*. *australis*-infected THP-1 derived macrophages. Consistent with the results from human PBMC-derived macrophages (Fig 1B), a significantly higher level of IL-1β was observed in *R*. *australis*-infected THP-1 derived macrophages compared to uninfected controls at 24 h p.i. (Fig 2A). Interestingly, *Rickettsia*-infected THP-1 derived macrophages treated with an inhibitor of caspase-1 produced a significantly less fold change in IL-1β compared to untreated controls (Fig 2B), suggesting that caspase-1 is critical for secretion of inflammasome-derived IL-1β in human macrophages upon rickettsial infection. Therefore, these results suggest that *R*. *australis* infects both human PBMC-derived macrophages and THP-1 cells, and promotes caspase-1-dependent secretion of IL-1β most likely via activating inflammasome pathway.

### *R*. *australis* activates inflammasome in mouse macrophages

**Fig 3:**
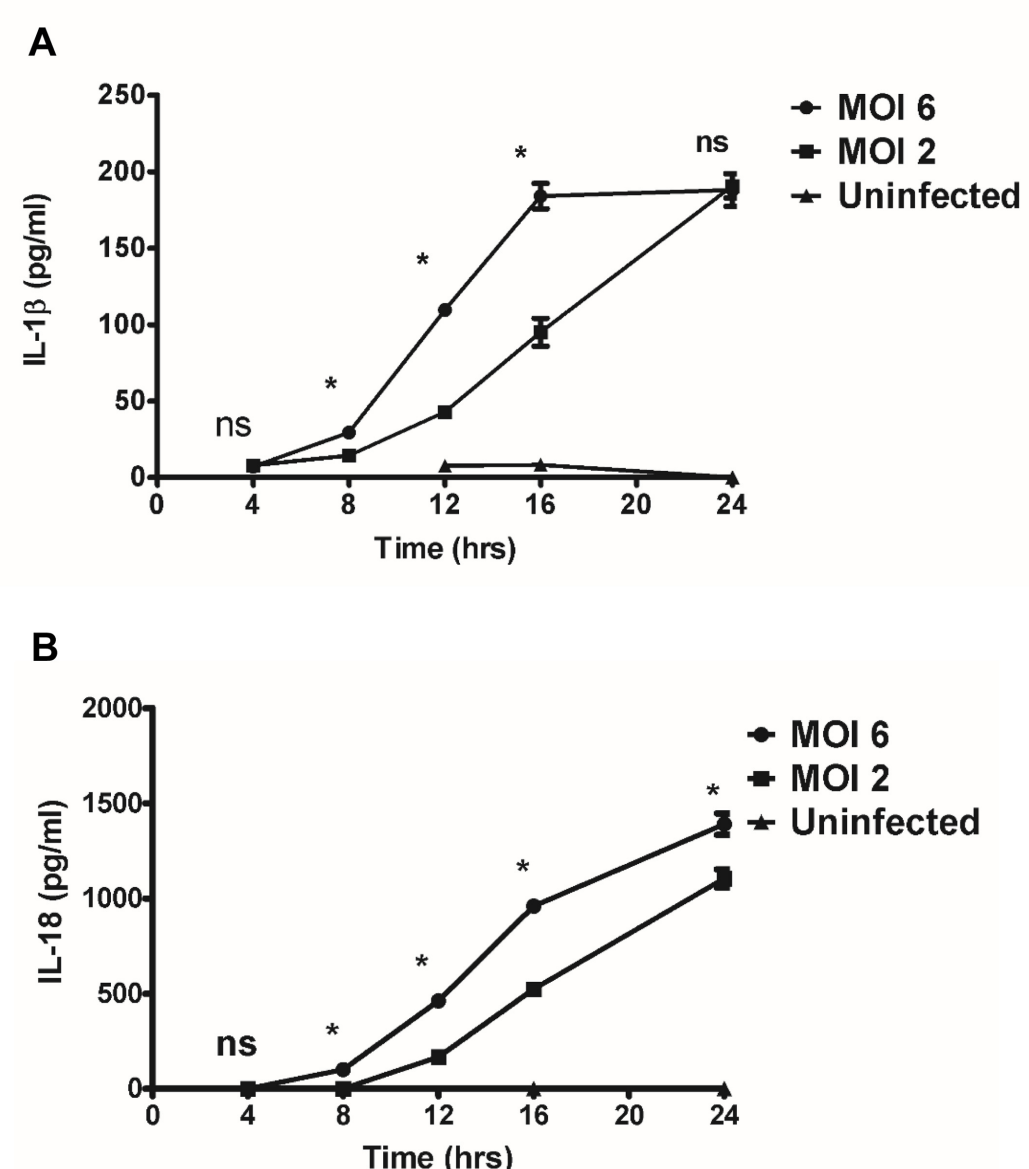
Kinetics and dose-dependent mechanisms of secretion of IL-1β and IL-18 by *Rickettsia*-infected BMMs. WT BMMs were isolated, cultivated, and infected with *R*. *australis* at MOI of 2 or 6. Cell culture supernatants were harvested at 4 hour intervals over 24 h p.i.. Secretion of IL-1β (A) and IL-18 (B) was determined by ELISA. Data represent two independent experiments with consistent results. Each experiment included at least 4 replicates. *, *p*<0.05; ns, not significantly different.

Activation of inflammasomes by microbes promotes the cleavage and maturation of pro-inflammatory cytokines IL-1β and IL-18 in a caspase-1/caspase-11-dependent manner. To investigate whether *R*. *australis* activates inflammasome in mouse macrophages, we first determined the secretion of inflammasome-derived IL-1 family cytokines including IL-1β and IL-18 by *R*. *australis*-infected mouse macrophages as well as investigating the kinetics- and dose-dependent mechanisms involved. As positive controls, WT BMMs stimulated with LPS plus ATP produced significantly higher levels of IL-1β and IL-18 compared to unstimulated controls. As shown in Fig 3A, *R*. *australis* induced significant secretion of IL-1β at 8 h p.i. upon infection at a high dose (MOI of 6) and at 12 h p.i. at a low dose (MOI of 2). The levels of IL-1β induced by a high dose of *R*. *australis* were significantly higher than those with the low dose infection at 8 h, 12 h and 16 h p.i.. Interestingly, although the production levels of IL-1β increased over time, there was no difference between the levels at high and low doses of infection at 24 h p.i. (Fig 3A). Very similar kinetics and dose-dependence of IL-18 production were observed in infected BMMs (Fig 3B) with the exception that a significant difference was observed between the levels at high and low doses after rickettsial infection at 24 h p.i.. Previous studies have demonstrated that rickettsiae induce negligible cytotoxicity in mouse peritoneal macrophages and the macrophage-like cell line, P388D1 [29]. In line with these previous studies, we did not find significantly reduced viability of mouse BMMs by trypan blue staining after infection with *R*. *australis* at 24 h p.i.. We also confirmed these results with LIVE/DEAD® Fixable Dead Cell Stain by flow cytometric analysis (S1 Fig).

**Fig 4:**
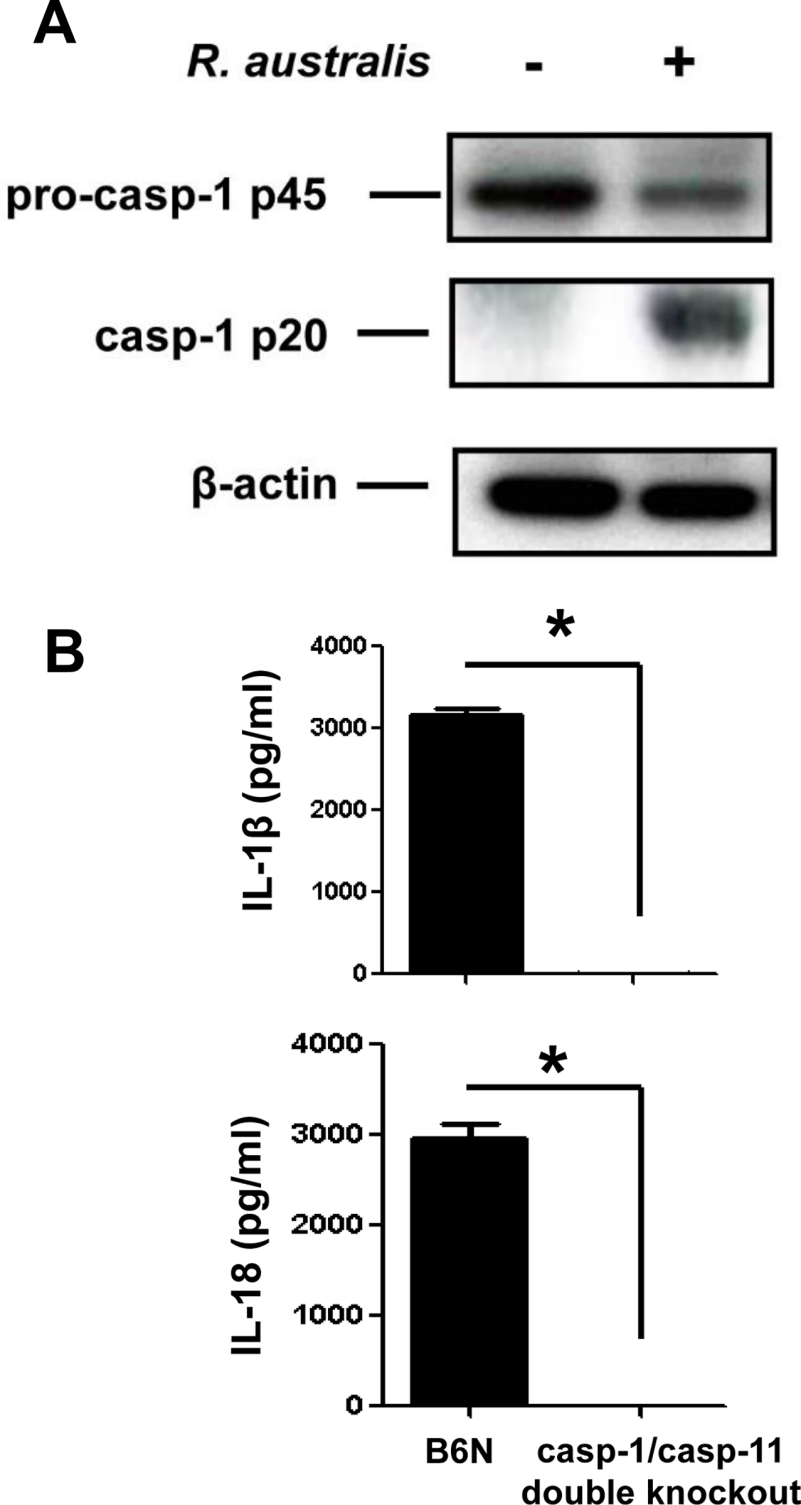
*R*. *australis* activated inflammasome in BMMs. A, WT BMMs were isolated, cultivated and infected with *R*. *australis* at an MOI of 10. At 24 h p.i., culture supernatant and cell lysates were collected. Cell lysates were processed for detection of activation of caspase-1. B, BMMs of B6N and caspase-1/11-double knockout mice were isolated and infected with *R*. *australis* as described above. The secretion levels of IL-1β and IL-18 were determined by ELISA. *, *p*<0.05.

To further determine whether the secretion of IL-1β and IL-18 upon rickettsial infection is mediated by inflammasome, we investigated the activation of caspase-1 and the role of casp-1/11 in this process. The cell lysates of uninfected and infected BMMs showed expression of pro-caspase-1 (p45) while activated caspase-1 (p20) was detected only in infected samples (Fig 4A). To confirm that the inflammasome pathway accounts for the production of cytokines such as IL-1β and IL-18 in *R*. *australis* -infected macrophages, we employed caspase-1/11-double knockout mice and the corresponding controls, B6N mice. B6N mice have the same genetic background as the caspase-1/11-double knockout mice. In response to *R*. *australis* infection, BMMs from B6N mice produced significant levels of IL-1β and IL-18 (Fig 4B). However, we did not detect any production of these cytokines in caspase-1/caspase-11-double knockout mice. These results confirmed that caspase-1 and/or caspase-11 are essential for production of IL-1β and IL-18, suggesting that *R*. *australis* activates inflammasome in mouse macrophages.

### ASC inflammasome is required for recognition of cytosolic *R*. *australis*

**Fig 5:**
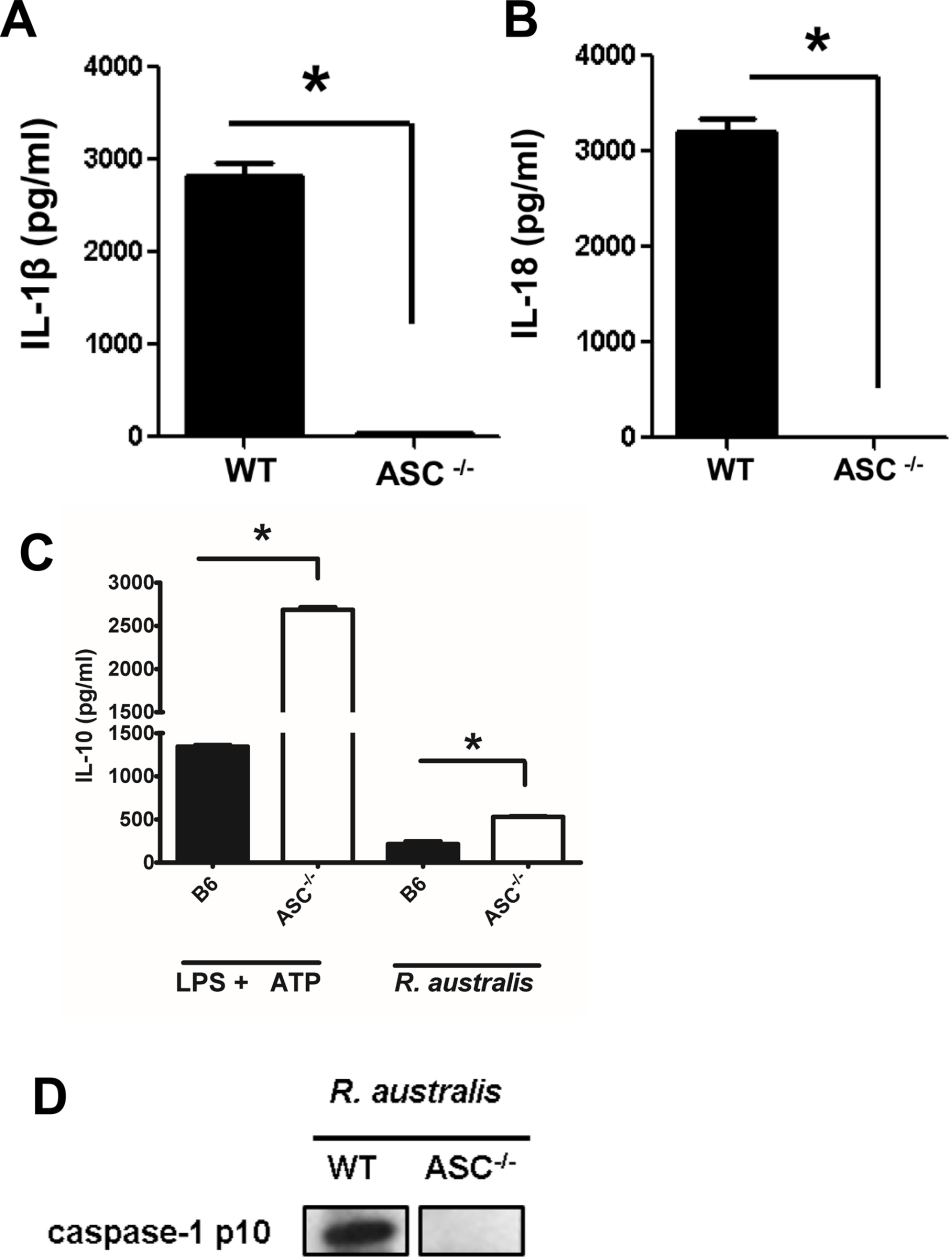
ASC-dependent inflammasome recognized cytosolic rickettsiae. BMMs of WT and ASC^-/-^ mice were isolated, cultivated, and infected with *R*. *australis* at an MOI of 10 or treated with LPS plus ATP as described in Materials and Methods. At 24 h p.i., the secretion of IL-1β (A), IL-18 (B) and IL-10 (C) was assayed by ELISA. D, Activation of caspase-1 was determined by immunoblotting detection of the active unit p10 in the processed supernatant of infected WT and ASC^-/-^ BMMs at 24 h p.i.. Data represent two independent experiments with consistent results. Each experiment included at least 4 replicates. *, *p*<0.05.

Next, we aimed to identify the NLRs involved in the recognition of *R*. *australis* in the host cell cytosol. Except NLRC4, most of the inflammasome NLRs signal through a critical adaptor protein, ASC. Upon rickettsial infection, BMMs of ASC^-/-^ mice failed to produce significant levels of IL-1β and IL-18 (Fig 5A and 5B). To exclude the possibility of unresponsiveness of ASC^-/-^ BMMs upon stimulation, we determined the production of IL-10, an inflammasome-independent cytokine. Interestingly, both *R*. *australis*-infected and LPS plus ATP-stimulated ASC^-/-^ BMMs produced significantly higher levels of IL-10 than WT BMMs (Fig 5C), suggesting that ASC^-/-^ BMMs were responsive to rickettsial infection. These results also exclude the possibility that the incapability of ASC^-/-^ BMMs to produce inflammasome-derived IL-1 family cytokines upon rickettsial infection was due to the failure of taking up *R*. *australis* intracellularly. To confirm that the abolished secretion of IL-1β and IL-18 in ASC^-/-^ BMM is not due to mechanisms other than inactivation of caspase-1, we determined the cleavage of caspase-1 in the supernatant of infected WT and ASC^-/-^ BMM by immunoblotting. As shown in Fig 5D, at 24 h p.i., activated caspase-1 p10 was only detected in infected WT but not ASC^-/-^ BMMs. Therefore, ASC, or ASC-dependent inflammasomes, were essential for the recognition of *R*. *australis* in the cytosol of mouse macrophages. Our results also suggest that ASC may suppress the production of IL-10 in response to infectious stimuli including rickettsial antigen.

### NLRP3 mediates the recognition of *R*. *australis* by inflammasome in BMMs

**Fig 6:**
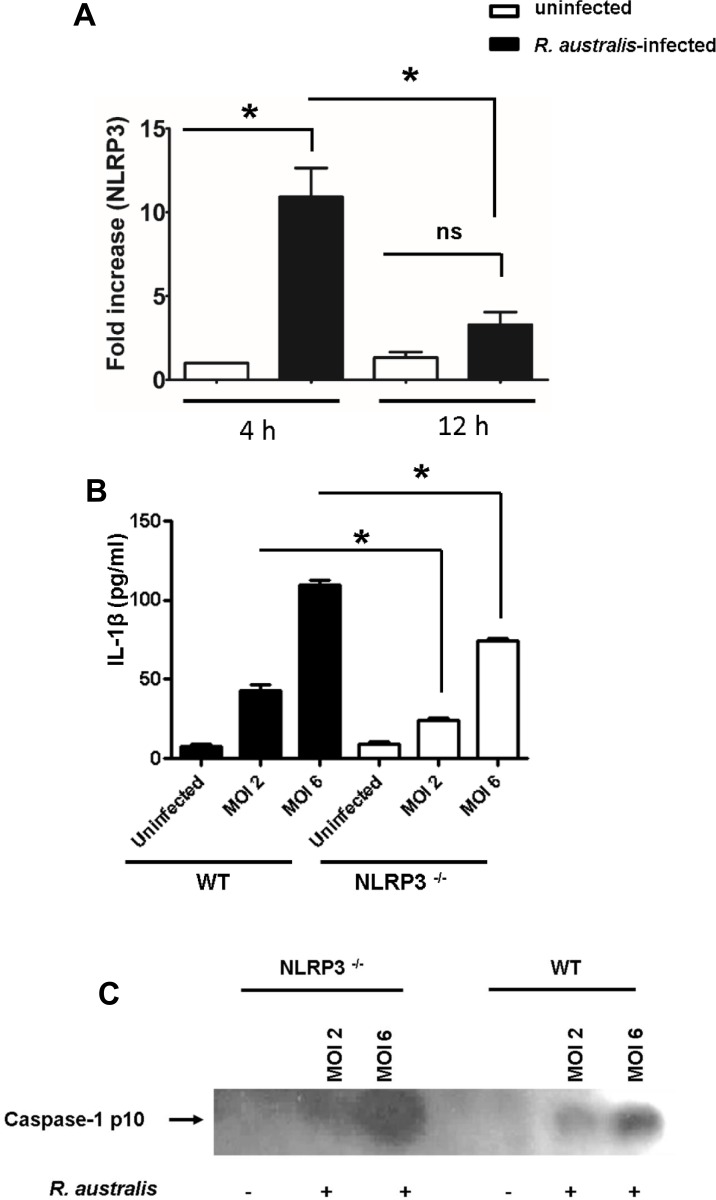
NLRP3 was involved in recognition of rickettsiae by inflammasome at the early stage of infection in BMMs. WT and NLRP3^-/-^ BMMs were isolated, cultivated, and infected with *R*. *australis* at MOIs of 6 and 2. The transcriptional levels of NLRP3 in WT BMMs at different time intervals of infection (at MOI of 6) were determined by RT-PCR as described in Materials and Methods (A). At 12 h (B) p.i., the secretion levels of IL-1β by WT and NLRP3^-/-^ BMMs were determined by ELISA. The cleavage of caspase-1 was determined by detection of the active unit p10 in the processed supernatant at 12 h p.i. (C). Data represent mean ± SD for at least 3 replicates each group. *, *p*<0.05 for a significant difference between WT and NLRP3^-/-^ mice; ns, not significantly different.

To further investigate the NLR inflammasome(s) responsible for recognition of rickettsiae in the cytosol, we first examined the transcriptional expression levels of NLRP3 in WT BMMs upon rickettsial infection. As early as 4 h p.i., infection with *R*. *australis* at an MOI of 6 significantly increased NLRP3 transcripts in WT macrophages (Fig 6A). As infection progressed, the quantity of NLRP3 mRNA progressively decreased at 12 h p.i. compared to 4 h p.i.. The transcriptional levels of NLRP3 were not significantly increased at 12 h p.i. compared to uninfected controls (Fig 6A). To further investigate whether NLRP3 is responsible for recognition of rickettsiae in the cytosol, we infected NLRP3^-/-^ and WT BMMs with *R*. *australis* at both high (MOI of 6) and low (MOI of 2) doses. At 12 h p.i., the secretion levels of IL-1β by NLRP3^-/-^ BMMs were significantly decreased compared to WT counterparts in response to both low and high doses of *R*. *australis* (Fig 6B). To further confirm that NLRP3 is involved in inflammasome activation by *R*. *australis*, we determined the cleavage of caspase-1 in the processed supernatant of WT and NLRP3^-/-^ BMMs by immunoblotting. As shown in Fig 6C, at 12 h p.i., activated caspase-1 p10 was detected in the supernatant of infected WT BMM. The density of caspase-1 p10 was correlated with the dose of rickettsial infection, suggesting a dose-dependent inflammasome activation mechanism. In line with Fig 6B, the cleaved caspase-1 was detected in NLRP3 ^-/-^ BMMs infected with *R*. *australis* at a high dose infection (Fig 6C). These results suggest that NLRP3 mediates the secretion of IL-1β by inflammasome pathway upon rickettsial infection and that there is an alternative NLRP3-independent pathway for inflammasome activation. These data also suggest that NLRP3 inflammasome contributes to recognition of *R*. *australis* in mouse macrophages. Thus, our results demonstrate that NLRP3 is activated by *R*. *australis* in BMMs.

### NLRP3 inflammasome contributes to the *in vivo* control of *R*. *australis* in spleen

To further explore the role of NLRP3 inflammasome in host defense against rickettsial infections *in vivo*, we measured rickettsial loads in infected tissues and survival of infected NLRP3^-/-^ and WT mice. Interestingly, the concentrations of *R*. *australis* in the spleen of NLRP3^-/-^ mice were significantly higher than those in WT mice on day 4 p.i., but not at day 2 p.i., suggesting that NLRP3 contributes to rickettsial elimination in spleen (S2 Fig). On days 2 and 4 p.i., rickettsial loads in liver and lung of NLRP3^-/-^ mice were not significantly different from those in infected WT mice (S2 Fig). Furthermore, compared to day 2 p.i., bacterial loads in tissues of NLRP3^-/-^ mice on day 4 p.i. were greater, particularly in spleen, suggesting that rickettsial infection progresses in NLRP3^-/-^ mice (S2 Fig). We did not find any significant difference in the survival of NLRP3^-/-^ and WT mice upon infection with *R*. *australis* at a dose of 2.8 × 10^5^ plaque-forming units (PFUs) (S3 Fig). Furthermore, histopathological analysis did not show any significant difference in inflammatory infiltrations in infected lung, liver and spleen of infected NLRP3^-/-^ and WT mice in either day 2 or day 4 p.i. (S4 Fig). These data suggest that the contribution of NLRP3 inflammasome to host control of *R*. *australis in vivo* is tissue- or cell type-specific. Taken together, these data suggest that NLRP3 inflammasome is not crucial to control rickettsial infection *in vivo* and only contributes to host control of rickettsiae in a tissue- or cell-specific mechanism.

## Discussion

In this study, we have demonstrated that cytosolic-replicating *R*. *australis* infects human primary and THP-1-derived macrophages, and induces the secretion of caspase-1-dependent cytokines, most likely through inflammasome pathway, which had never been reported previously. *R*. *australis* activated inflammasome in mouse macrophages via time- and dose-dependent mechanisms. ASC-dependent inflammasomes were responsible for recognition of *R*. *australis* in host cytosol while NLRP3 inflammasome significantly contributed to this process. The *in vivo* role of NLRP3 inflammasome in host immune responses to *R*. *australis* was tissue-specific as evidenced by significantly increased bacterial loads in spleen, but not liver and lung, of NLRP3^-/-^ mice compared to WT mice. More importantly, for the first time, we demonstrated that *R*. *australis* activated inflammasome in human macrophages with kinetics that differed from mouse macrophages. Our findings have provided novel knowledge of the mechanisms by which the host immune surveillance system interacts with *Rickettsia* via macrophages.

*Rickettsia australis* activated ASC-dependent inflammasome in murine BMMs as indicated by the following evidence: 1) Secretion of IL-1β and IL-18 upon infection was completely abrogated in cells deficient in ASC and caspase-1/caspase-11-double knockout cells (Figs 4 and 5); 2) *Rickettsia australis* infection induced cleavage of caspase-1 in the cell lysates and supernatant (Figs 4A, 5D and 6C). Although neutrophil-dependent, inflammasome-independent processing of IL-1β has been described recently [30], our current data excluded the possibility of inflammasome-independent processing and secretion of IL-1β and IL-18 and demonstrated inflammasome activation by *R*. *australis* in macrophages. ASC serves as the essential adaptor molecule for several NLRs including NLRP1, NLRP3 and absent in melanoma 2 (AIM2) [31, 32]. Our results showed that NLRP3 contributed significantly to the activation of inflammasome by *R*. *australis* in BMMs. Future investigations are required to reveal the upstream signals mediating the activation of NLRP3 inflammasome by *R*. *australis*, such as potassium efflux [33, 34], lysosomal degradation [35], and ROS production [36]. Interestingly, we also found that a significant level of inflammasome-derived IL-1β secretion was NLRP3-independent (Fig 6B), particularly at a high dose of infection, which suggests that ASC-dependent NLR inflammasomes other than NLRP3, potentially NLRP1 and/or AIM2, coordinate with NLRP3 or also play a significant role in the recognition of cytosolic *R*. *australis*. Furthermore, we did not find significant difference in IL-1β secretion by infected BMMs of NLRP3^-/-^ mice compared to WT mice at 24 h p.i., a time at which the levels of IL-1β secretion reached a peak in macrophages of WT mice (Fig 3A). The differential contributions of NLRP3 to inflammasome activation by *R*. *australis* at early versus late time points are likely explained by two possibilities: 1) Inflammasomes other than NLRP3 play a major role in recognizing *R*. *australis* and the related danger signals at the late stage of infection; 2) NLRP3 inflammasome is down-regulated by other immune mechanisms such as caspase-11, autophagy, or cytokines specifically suppressive for NLRP3. These *in vitro* findings may account for the dispensable role of NLRP3 inflammasome in host control of *R*. *australis in vivo*. Although it is less likely that a higher dose of *R*. *australis* could lead to differential host susceptibilities of NLRP3^-/-^ mice compared to WT mice (S3 Fig), our data clearly illustrated the significant contribution of NLRP3 inflammasome to host defense in a tissue-specific manner (S2 Fig). Considering the different proportions of cell types in spleen compared to liver and lung, *R*. *australis* may mainly activate NLRP3 inflammasome in leukocytes such as macrophages. Interestingly, we found significantly enhanced host susceptibilities and increased rickettsial loads in tissues of mice deficient in ASC during rickettsial infection compared to WT mice in our preliminary *in vivo* studies. Our previous studies have clearly demonstrated that the *in vivo* production of IL-10 in murine models of fatal rickettsioses is associated with the severity of disease [37]. As shown in Fig 5C, ASC significantly suppressed the secretion of IL-10 by *R*. *australis*-infected macrophages, implying that ASC may contribute to host resistance against rickettsiae. Although further investigations are required to completely understand how inflammasome contributes to host immunity *in vivo* against these intracellular bacteria, our data suggest that ASC/NLRP3 inflammasome plays a role in host defense.

Among the different types of microbes, cytosolic bacteria are uniquely useful for investigating inflammasome activation mechanisms due to their biological characteristics and the site where inflammasomes initiate their recognition of microbes or microbial products. Inflammasome activation by rickettsial infection in macrophages was both time-and dose-dependent. Distinct from other facultatively cytosolic bacteria including *Listeria*, *Shigella*, *Burkholderia* and *Francisella*, rickettsiae are obligately cytosolic bacteria which quickly escape phagosomal vacuoles and replicate within the cytosol of host cells including macrophages. Mouse macrophages secrete IL-1β and IL-18 in response to *Listeria* at 5 h p.i. [38], *Shigella* at 6 h p.i. [39], *Burkholderia* at 4 h p.i.[40], and *Francisella* at 5 h p.i.[41]. Our data suggest that the kinetics and possibly the mechanisms of inflammasome activation by *R*. *australis* are distinct from other cytosolic bacteria. In response to *R*. *australis*, mouse macrophages secrete IL-1β and IL-18 as late as at 8 h p.i. after a high dose of infection and at 12 h after a low dose of infection. The levels of these inflammasome-derived cytokines increased progressively as the infection progressed and reached a peak at 24 h p.i. regardless of the dose. The delayed activation of inflammasome by *R*. *australis* in mouse macrophages compared with several facultatively cytosolic bacteria mentioned above suggests that this intracellular bacterium may initiate an evasion mechanism to escape inflammasome assembly at the early stage of infection. The dose-independent secretion of IL-1β at 24 h p.i. by infected mouse macrophages suggests that inflammasomes responsible for recognizing these intracellular bacteria at the late stage of infection are very sensitive to the activation of ligand(s) generated during rickettsial infection, and could be an ideal candidate for vaccine development targeting inflammasome activation in the future.

While we have shown that *R*. *australis* activated caspase-1-dependent inflammasome in both murine and human macrophages, it remains unclear whether canonical or noncanonical inflammasomes are activated by rickettsiae. Kayagaki et al. demonstrated that the non-canonical inflammasome pathway engages caspase-11 to activate caspase-1 and the subsequent release of IL-1β and IL-18 [42]. *Ehrlichia*, another obligately intracellular bacterial species closely related to rickettsiae, triggers cleavage of caspase-1 and IL-18 secretion in BMMs [43]. Interestingly, type I interferon signaling promotes host susceptibility to fatal ehrlichiosis potentially via activation of non-canonical inflammasomes [43]. Thus, it is an attractive hypothesis that caspase-11 mediates caspase-1 activation, which further processes IL-1β secretion in *R*. *australis*-infected murine macrophages. Recently several intracellular bacterial pathogens, including *Legionella pneumophila*, *Yersinia pseudotuberculosis*, and *Salmonella enterica* serovar Typhimurium *(S*. *Typhimurium)*, were reported to activate both canonical caspase-1-dependent and non-canonical caspase-1-independent inflammasomes in primary human macrophages [44]. Considering the fact that we have not examined caspase-11-dependent inflammatory cell death, pyroptosis, in infected human macrophages, our data suggest that *R*. *australis* at least activates canonical inflammasome in human macrophages. Furthermore, NLRP3 inflammasome has been described to be involved in both canonical and non-canonical inflammasome pathways [42, 45]. Therefore, our future studies will investigate whether the canonical or non-canoncial pathway accounts for NLRP3 activation by rickettsial infection.

In agreement with our data in murine macrophages, *R*. *australis* not only infects both human primary and THP-1 derived macrophages, but also activates the inflammasome in human macrophages. It is worth noting that: 1) The kinetics of secretion of IL-1β by *R*. *australis* -infected macrophages may differ from IL-18. We observed a significant difference in secretion levels of IL-18, but not IL-1β, in mouse macrophages at high and low doses after rickettsial infection at 24 h p.i. (Fig 3). Furthermore, *R*. *australis* initiated significant secretion of IL-1β, but not IL-18, as early as 3 h p.i. in human primary macrophages (Fig 1); 2) Induction of IL-1β by *R*. *australis* in human primary macrophages (3 h p.i.) occurred much earlier than in mouse macrophages (8 h p.i.) (Figs 1B and 3A). These findings suggest that: 1) The inflammasome pathways mediating secretion of IL-1β are likely different compared to IL-18 during rickettsial infection; 2) Activation mechanisms of inflammasome by *R*. *australis* in human macrophages are potentially different from the mouse counterparts. Future studies focusing on these interesting points will not only provide information critical for further understanding the pathogenesis of severe rickettsioses but also will enable us to uncover new mechanisms involved in activation of inflammasomes.

The present study investigated the essential and dispensable components in the inflammasome pathway in mouse and human macrophages, which have never been previously examined during rickettsial infection. *Rickettsia australis* activated ASC-dependent inflammasomes in which NLRP3 contributed significantly to recognition of the bacteria. Our data suggest inflammasomes other than NLRP3 might play a critical role in the cytosolic surveillance system at the late stage of rickettsial infection. Our investigations also point out the potentially important role of macrophages in human rickettsioses.

## Supporting Information

S1 Fig *R*. *australis* infection did not cause significant cell death in mouse macrophages. BMMs were isolated and infected with *R*. *australis*. At 24 h p.i., uninfected and infected macrophage viability was examined as described in Materials and Methods.
(TIF)

S2 Fig NLRP3 inflammasome contributed to the *in vivo* control of rickettsial infection in spleen. NLRP3^-/-^ and WT mice were infected i.v. with *R*. *australis* at a dose of 1 × 10^5^ PFU per mouse. On days 2 and 4 p.i., bacterial loads in spleen, liver and lung of infected mice were determined by quantitative real-time PCR. Results are means ± SE of data from two independent experiments with consistent results, where each experimental group included 4 mice. *, *p*<0.05.
(TIF)

S3 Fig Host susceptibility of NLRP3^-/-^ mice to infection with *R*. *australis* was not significantly different compared to WT mice. NLRP3^-/-^ and WT mice were infected i.v. with *R*. *australis* at a dose of 2.8 × 10^5^ PFUs per mouse. Mice were monitored daily until day 20 p.i..
(TIF)

S4 Fig Histopathological analysis did not show any significant difference in inflammatory infiltrations in lung, liver and spleen of infected WT and NLRP3^-/-^ mice. NLRP3^-/-^ and WT mice were infected i.v. with *R*. *australis* at a dose of 1 × 10^5^ PFU per mouse. On days 2 and 4 p.i., mice were sacrificed and tissues were processed for histopathological analysis.
(TIF)
